# Application value of multi-disciplinary collaborative diagnosis and treatment combined with CBL teaching model in gynecological oncology practice teaching

**DOI:** 10.3389/fmed.2024.1468256

**Published:** 2025-01-08

**Authors:** Fengrong Wen, Julan Mo, Renxiang Li

**Affiliations:** ^1^College of Nursing, Zhaoqing Medical College, Zhaoqing, China; ^2^Department of Gynaecology and Obstetrics, Zhaoqing Maternal and Child Health Care Hospital, Zhaoqing, China; ^3^Department of Neurosurgery, Zhaoqing Gaoyao District People's Hospital, Zhaoqing, China

**Keywords:** multidisciplinary collaborative diagnosis and treatment, case-based learning method, gynecological tumors, internship teaching, teaching model

## Abstract

**Background:**

To explore the application value of multi-disciplinary collaborative diagnosis (MDT) and treatment combined with the case-based learning (CBL) teaching method based on real clinical cases in gynecological malignant tumor practice teaching.

**Methods:**

A total of 120 clinical students who were interning in the Department of Gynecology in our hospital from January 2022 to June 2023 were selected and divided into a research group (*n* = 60) and a control group (*n* = 60) according to the random number table method. The research group adopted a MDT combined with the CBL teaching model, while the control group followed a traditional teaching model. After the two-month internship, the teaching faculty completed a self-evaluation form, and the students jointly evaluated the teaching effect through an exit assessment, which included basic theory, clinical skills, and case assessment. Additionally, a questionnaire survey was conducted to evaluate the student’s recognition of the teaching model and collect their opinions and suggestions.

**Results:**

The research group showed significantly higher scores in basic theoretical knowledge, clinical skills, and case analysis assessments compared to the control group (*p* < 0.05). The questionnaire survey results indicated that the research group outperformed the control group in knowledge acquisition, learning initiative, learning interest, clinical analysis ability, clinical diagnosis, treatment thinking, teamwork ability, literature retrieval, and reading ability, and clinical language expression ability (*p* < 0.05). Students’ feedback suggested increasing doctor-patient communication time and improving the doctor-patient communication skills.

**Conclusion:**

MDT combined with the CBL teaching model based on real clinical cases can effectively foster autonomous learning, enhance the application of basic theoretical knowledge, and improves the quality of clinical teaching in gynecology. This method is worthy of promotion in clinical teaching.

## 1 Background

With the continuous advancement of medical technology and the improvement of public health awareness, women’s health issues have received unprecedented attention and importance. According to recent global health statistics, gynecological cancers such as ovarian, cervical, and endometrial cancers are among the leading causes of cancer-related deaths in women worldwide (1). The rising incidence of various tumors, including gynecological malignancies such as ovarian cancer, cervical cancer, and endometrial cancer, significantly contributes to global morbidity and mortality. These diseases are not only diverse but also involve a wide range of diseases, presenting significant challenges in diagnosis and treatment (2). Faced with the complexity of medical and surgical treatment of gynecological malignancies, clinical gynecologists need to have higher professional knowledge and skills to ensure that they can accurately identify symptoms, develop effective treatment plans, and provide high-quality diagnosis and treatment services (3).

As medical education evolves toward a competency-based, student-performance-focused curriculum, the demand for teaching models is increasing under the new situation, and standardized medical education is increasingly valued by the world (4). At present, traditional teaching methods are still the main method in medical and clinical teaching. This is necessary and effective for popularizing important knowledge and concepts. Because it often has a large audience, the traditional lecture teaching method is indeed the most economical and effective theoretical teaching method (5). However, the traditional teaching model is dominated by clinical teachers, who often adopt a cramming teaching method, where teachers actively transfer theoretical knowledge, while students (in most cases) passively listen, annotate and accept the content presented by teachers, that is, knowledge is instilled into students in a one-way manner (6).

However, traditional medical training falls short in equipping students with the interdisciplinary competencies needed in modern medicine. Several studies indicate that traditional lecture-based approaches lack the engagement and practical application required for complex fields such as gynecological oncology, where both interdisciplinary knowledge and clinical skills are essential (7–9). The traditional medical education model struggles to adapt to the requirements of contemporary medical practice (10, 11). Teaching hospitals, therefore, play a crucial role in hands-on clinical training of interns from medical universities, as they provide exposure to real-world medical scenarios and patient interactions (12). Consequently, there is a pressing need to explore innovative teaching models that enhance engagement, clinical reasoning, and practical skills, ultimately improving the quality of medical education.

Case-based learning (CBL) is a teaching strategy that has gained significant acclaim both domestically and internationally in recent years (13). CBL’s effectiveness has been documented across various disciplines, as it allows students to apply theoretical knowledge in realistic clinical contexts, promoting deep learning and enhancing clinical reasoning skills (14–16). Its core concept involves constructing learning scenarios through in-depth analysis of specific cases. This method typically places students in simulated environments akin to real clinical settings, allowing them to deepen their understanding and mastery of knowledge through problem-solving and addressing challenges. CBL encourages students to actively explore and discover new knowledge points, fostering critical thinking and innovation (17–19). The flexibility and student-centered approach of CBL also cater to the diverse learning needs of medical students, making it a preferred teaching model in various medical curricula. Through this teaching model, students become acquainted with complex cases across various medical fields and learn to apply theoretical knowledge in practice, thereby transforming and enhancing their knowledge.

Multidisciplinary treatment (MDT) of tumors involves systematic discussions by experts from multiple disciplines, based on the patient’s condition and tumor characteristics, to scientifically integrate various diagnosis and treatment measures and formulate the most appropriate treatment plan (20). MDT has become the preferred treatment model for gynecological malignancies (21). The MDT model enhances patient care outcomes and has demonstrated improved survival rates in gynecological oncology by leveraging insights from fields such as radiology, pathology, surgery, and pharmacology (22, 23). In clinical teaching, the MDT model was initially implemented in tumor specialties but is typically integrated into CBL or problem-based learning rather than used alone. Studies have shown that combining MDT with CBL can provide students with a holistic view of patient care, equipping them with the multidisciplinary perspective needed in today’s collaborative healthcare environment (17, 24). A review of the literature revealed that the CBL method has been applied in undergraduate and graduate medical education (25), but no systematic scope review has thoroughly explored the effectiveness of the MDT model combined with CBL teaching (26).

In this study, we employed the MDT combined with the CBL teaching model in the clinical teaching of gynecological tumors. We then analyzed the application value of this teaching model in gynecological medicine education, aiming to cultivate medical talents with a solid theoretical foundation and practical skills through high-quality education and training.

## 2 Methods

### 2.1 Sample

120 clinical students who were doing internships in the gynecology department of our hospital from January 2022 to June 2023 were selected. The internship period was 2 months and they were divided into a research group (*n* = 60) and a control group (*n* = 60) according to the random number table method. The above patients and their families were informed of the relevant contents of this study, voluntarily signed the consent form, and actively cooperated with the research activities. The study protocol was approved by the hospital ethics committee, ensuring adherence to ethical standards in educational research.

Inclusion criteria: (a) fully participate in the research intervention during the entire internship period; (b) students and their families sign the informed consent form.

Exclusion criteria: (a) not fully participate in the research intervention during the entire internship period; (b) disagree to sign the informed consent form.

### 2.2 Procedure

#### 2.2.1 Control group (traditional teaching mode)

The control group adopted the traditional teaching mode: according to the requirements of the obstetrics and gynecology teaching syllabus, the focus was on the theoretical knowledge teaching of students, with the lecturer as the leading position. The main contents of the lecture included the concepts of various malignant tumors, the selection of diagnosis and treatment plans, etc. Typical cases of gynecological malignancies were presented, and multimedia resources were used for concentrated lectures and explanations. An interactive questioning session between teacher and student was added at the end, and students were encouraged to review after class.

#### 2.2.2 Research group (MDT combined with CBL teaching mode)

The research group adopted the MDT combined with CBL teaching mode.

##### 2.2.2.1 Case preparation and lesson design

The teacher formulated specific cases based on the textbook syllabus and clinical practice, focusing mainly on obstetrics, gynecology, and common diseases. The teaching plan was student-centered and teacher-led. The teacher prepared appropriate teaching cases and related questions according to the students’ situation and made PowerPoint presentations. Guided by key points from the cases, the teacher meticulously prepared lessons, incorporating relevant frontier nursing knowledge.

##### 2.2.2.2 Teaching process

During the teaching process, the host teacher used pre-set case questions to stimulate students’ thinking and curiosity. Students were divided into groups of 10 based on seating proximity, with each group centered around a case. Using the teaching syllabus and typical cases, students independently consulted literature, presented their views, and discussed problems in class. Discussion time was limited to 10 min. For instance, students might be asked, “How would you prioritize diagnostic procedures for a suspected case of ovarian cancer?” or “What considerations are necessary when selecting a treatment plan for a patient with cervical cancer?” The teacher engaged with student groups to understand different problem-solving approaches and thinking patterns. After gathering opinions from different groups, the teacher guided students to answer difficult questions by consulting relevant domestic and international literature.

##### 2.2.2.3 Group presentation and feedback

After the discussion phase, each group selected a representative to present their findings. The teacher provided immediate feedback and adjusted the class rhythm to ensure the completeness and order of the teaching content. To assess comprehension, questions aligned with the syllabus were set up as multiple-choice or voting questions. An example multiple-choice question included, “Which diagnostic test is considered most reliable for early detection of cervical cancer?” This approach comprehensively evaluated students’ understanding and engagement. Relevant questions were set according to the syllabus and case, conducted as multiple-choice questions or voting. This approach helped to comprehensively assess students’ understanding.

##### 2.2.2.4 Multidisciplinary involvement

Based on the case characteristics, teachers from various disciplines were invited to define and explain knowledge points related to their specialties. For instance:

###### 2.2.2.4.1 Inspection discipline

Focused on project principles, reasonable selection, specimen collection precautions, result interpretation, potential interference factors, and prevention of false positives and negatives.

###### 2.2.2.4.2 Imaging discipline

Interpreted imaging characteristics, compared different imaging technologies, and discussed “same disease, different images” and “different diseases, same images.”

###### 2.2.2.4.3 Clinical pharmacy

Covered the mechanism of action, pharmacokinetics, pharmacodynamics, adverse reactions, drug selection, and differences among drugs of the same type.

###### 2.2.2.4.4 Nursing discipline

Addressed nursing issues, risk assessment, dietary guidance, and life guidance.

##### 2.2.2.5 Summary and evaluation

Finally, the presiding teacher provided a systematic summary of the case diagnosis and treatment, and commented on each group’s pre-class literature review, discussion, and representative presentations, highlighting the strengths and weaknesses. The feedback aimed to reinforce accurate clinical reasoning and correct misunderstandings identified during the presentations.

After the two groups of internships were completed, the teachers filled out a self-evaluation form, and the students jointly evaluated the teaching effectiveness through an exit assessment that included basic theory, clinical skills, and case assessment. At the same time, a questionnaire survey was conducted to investigate students’ recognition of the teaching model and to collect students’ opinions and suggestions. The questionnaire was developed by our research team for the purposes of this research (Supplementary File S1). Example questions from the questionnaire included: “How would you rate your ability to apply theoretical knowledge in clinical practice after this training?” and “Did this teaching model increase your interest in learning gynecological oncology?”

### 2.3 Observation indicators

All 120 interns spent 2 months in a gynecological oncology internship. When they left the department, the instructors and all trainees were evaluated. The methods are as follows:

#### 2.3.1 Teachers’ evaluation of teaching effectiveness

The instructors were asked to self-evaluate their teaching effectiveness using the teacher self-evaluation form.

#### 2.3.2 Intern teaching effectiveness evaluation

The interns’ teaching effectiveness was evaluated using the proposed exit examination paper, which included basic theory, clinical skills, and case assessment. The basic theory was the knowledge and diagnosis and treatment theory related to gynecological oncology, which was assessed using multiple-choice questions, fill-in-the-blank questions, term explanations, and questions and answers; clinical skills included history writing, physical examination, and gynecological specialist examinations, and case analysis selected two typical cases of gynecological malignant tumors. The double-blind method was used, and the judges were not among the clinical instructors in this study; all scores were in percentage.

#### 2.3.3 Interns’ recognition of the teaching model and collection of feedback

The questionnaire survey was conducted and filled out anonymously by students. The content included knowledge acquisition ability, learning initiative, learning interest, clinical integration, clinical analysis ability, clinical diagnosis and treatment thinking, teamwork ability, literature retrieval and reading ability, doctor-patient communication ability, and clinical language expression ability. Examples of survey items included: “Rate your ability to retrieve and interpret relevant medical literature” and “How has this model influenced your doctor-patient communication skills?”

### 2.4 Data analysis

After the analysis and summary, the relevant data in the study were entered into SPSS 26.0 statistical software for processing. The counting data between groups were expressed as “%,” and the measurement data consistent with the normal distribution were expressed by (*x* ± *s*). The former obtained the results by chi-square, and the latter obtained the results by *t*-test. If the difference between the test data was statistically significant, it was expressed as *p* < 0.05.

## 3 Results

### 3.1 Study sample

The comparison of general information between the two groups of interns showed no significant differences in gender, age, and pre-entry grades (Table 1).

**Table 1:** General information of the two groups of interns.

Item	Research group (*n* = 60)	Control group (*n* = 60)	*p*
Men/women (*n*)	32/28	31/29	0.855
Average age (years)	23.63 ± 0.74	23.47 ± 0.68	0.199
Pre-entry grades (points)	76.78 ± 11.91	76.42 ± 12.00	0.867

### 3.2 Teachers’ evaluation of teaching effect

After the two groups of teachers completed their teaching, the effects of different teaching modes were compared (Table 2). Overall, the students’ theoretical system and memory knowledge point construction, pre-class preparation, class discussion, and students’ practical ability under the new mode of the research group were better than those under the traditional mode of teaching, and the workload of teachers in the lesson preparation stage was also greater than that of traditional teaching. Compared with the control group, the teachers’ knowledge and abilities were significantly improved (*p* < 0.05).

**Table 2:** Teachers’ self-evaluation of teaching effectiveness with different teaching modes.

Question	Research group (*n* = 60)	Control group (*n* = 60)	*χ* ^2^	*p*
Is it helpful for students to build a theoretical system and memorize knowledge points?	53 (88.33%)	35 (58.33%)	13.807	<0.001
Are students able to complete their pre-class preparation as expected?	41 (68.33%)	30 (50.00%)	4.174	0.041
Can you fully participate in class discussions?	42 (70.00)	30 (50.00)	9.425	0.002
Does the lesson preparation process significantly increase teachers’ workload?	52 (86.67)	36 (60.00)	10.909	0.001
Is it helpful for improving teachers’ own knowledge and abilities?	50 (83.33)	35 (58.33)	9.076	0.003
Has there been improvement in students’ practical ability?	52 (86.67)	37 (61.67)	9.786	0.002

### 3.3 Evaluation of intern teaching effectiveness

**Figure 1:**
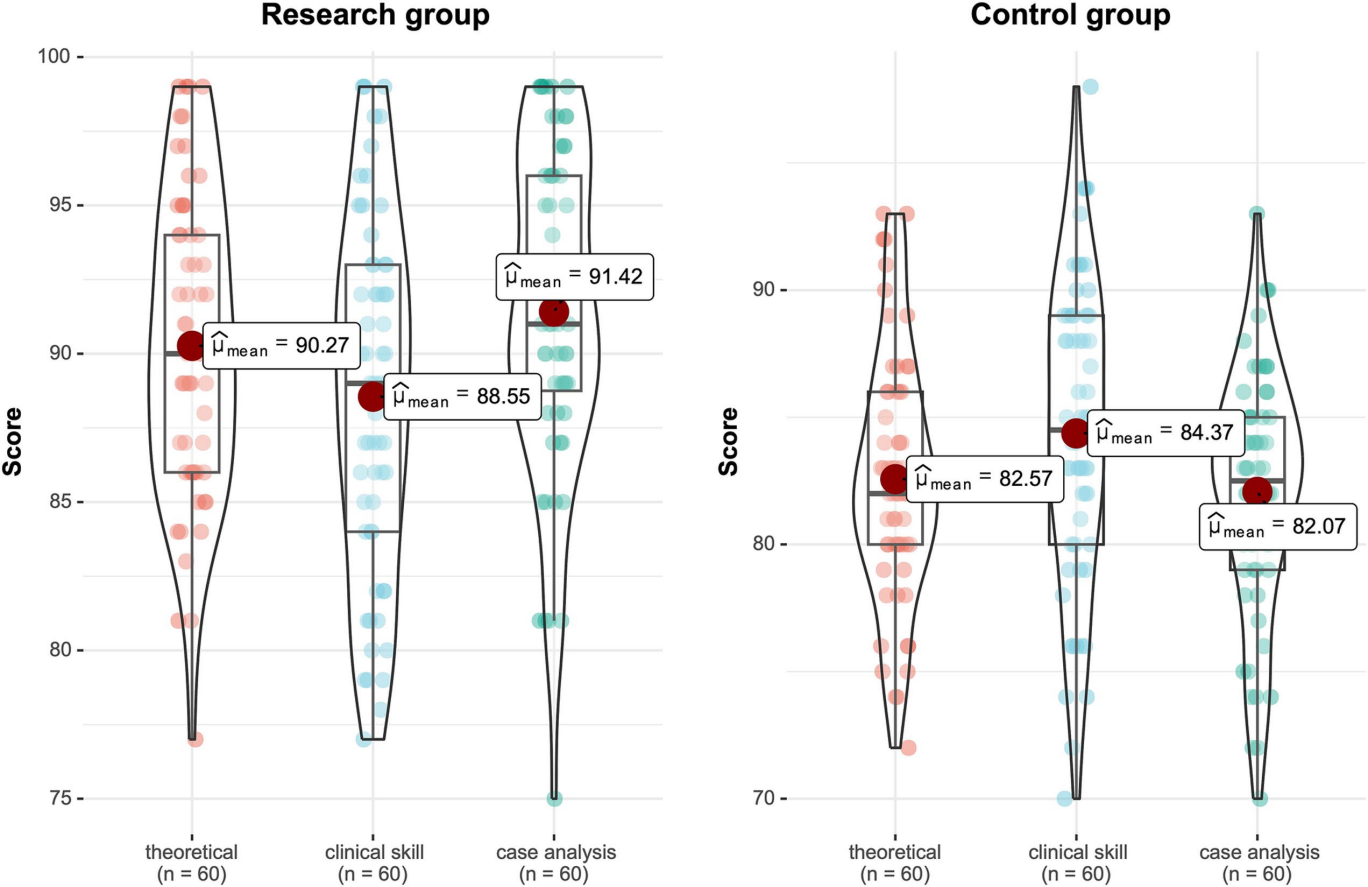
Violin plot of the two groups of interns’ final assessment results.

After the two-month teaching period, the teaching interns were evaluated using the prepared exit examination papers. The comparison results showed that the research group was significantly higher than the control group in terms of basic theoretical knowledge, clinical skills, and case analysis. The difference is statistically significant (*p* < 0.05) (Table 3 and Figure 1).

**Table 3:** The results of the two groups of interns’ final examination.

Group	*n*	Basic theoretical knowledge	Clinical skills	Case analysis
Research group	60	90.27 ± 5.11	88.55 ± 5.97	91.42 ± 5.50
Control group	60	82.57 ± 4.89	84.37 ± 6.04	82.07 ± 4.80
*t*		8.341	3.815	9.924
*p*		<0.001	<0.001	<0.001

### 3.4 Interns’ recognition of the teaching model

After the two-month teaching, the results of the questionnaire survey on the interns were analyzed. The results showed that the research group was superior to the control group in terms of knowledge acquisition ability, learning initiative, learning interest, clinical integration, clinical analysis ability, clinical diagnosis and treatment thinking, teamwork ability, literature retrieval and reading ability, doctor-patient communication ability, and clinical language expression ability. The difference was statistically significant (*p* < 0.05) (Table 4). The students’ opinions and suggestions mainly included appropriately increasing the doctor-patient communication time and improving the doctor-patient communication skills.

**Table 4:** Questionnaire survey results on the teaching identity of the two groups of interns.

Investigate subject	Research group (*n* = 60)	Control group (*n* = 60)	*χ* ^2^	*p*
Knowledge acquisition ability	53 (88.33)	39 (65.00)	9.130	0.003
Improve learning initiative	49 (81.67)	32 (53.33)	4.385	0.036
Stimulate interest in learning	53 (88.33)	37 (61.67)	11.378	0.001
Increase clinical integration	58 (96.67)	31 (51.67)	31.707	<0.001
Providing clinical analysis capabilities	49 (81.67)	34 (56.67)	8.792	0.003
Enhance clinical diagnosis and treatment thinking	55 (91.67)	40 (66.67)	11.368	0.001
Promote teamwork skills	51 (85.00)	36 (60.00)	9.404	0.002
Improve literature search and reading skills	46 (76.67)	28 (46.67)	11.422	0.001
Improve doctor-patient communication skills	45 (75.00)	32 (53.33)	6.125	0.013
Optimizing clinical language expression skills	47 (78.33)	32 (53.33)	8.336	0.004

### 3.5 Case study example and specific skills assessed

To further illustrate the application of the MDT combined with CBL teaching model in gynecological oncology training, we provide two specific examples of the types of skills assessed and a representative case study used during the teaching sessions which could support a practical understanding of the teaching model.

In the final assessment of the research group, three core competencies were evaluated: fundamental theoretical knowledge, clinical skills, and case analysis. Below are specific skills evaluated within each category:

#### 3.5.1 Fundamental theoretical knowledge

Mastery of diagnostic criteria and staging for common gynecologic cancers, such as ovarian, cervical, and endometrial cancers. Knowledge of standard treatment protocols, including the indications for surgery, chemotherapy, and radiotherapy. Understanding of pathophysiology related to gynecologic malignancies.

#### 3.5.2 Clinical skills

##### 3.5.2.1 Patient history and communication

Ability to conduct a comprehensive patient history with a respectful and empathetic approach.

##### 3.5.2.2 Physical examination

Proficiency in performing pelvic exams and recognizing key physical signs relevant to gynecologic oncology.

##### 3.5.2.3 Diagnostic reasoning

Competence in interpreting laboratory results (e.g., CA-125 levels) and imaging studies (e.g., ultrasound, CT scans) to formulate an accurate diagnosis.

#### 3.5.3 Case analysis and multidisciplinary decision-making

##### 3.5.3.1 Differential diagnosis

Ability to generate and justify a list of potential diagnoses based on patient symptoms and clinical findings.

##### 3.5.3.2 Treatment planning

Development of a comprehensive, individualized treatment plan that integrates multidisciplinary insights and considers patient preferences.

##### 3.5.3.3 Ethical consideration and reflection

Engagement in critical thinking regarding ethical aspects, such as patient autonomy and informed consent.

#### 3.5.4 Example case study: suspected ovarian cancer

##### 3.5.4.1 Patient presentation

A 52-year-old postmenopausal female presents with complaints of abdominal bloating, early satiety, and pelvic pain. She has a family history of breast and ovarian cancer, and a recent pelvic exam reveals a palpable adnexal mass and presence of ascites.

#### 3.5.5 Guided questions and teaching points

##### 3.5.5.1 Differential diagnosis and diagnostic process

Question: “What are the main differential diagnoses to consider, and what initial diagnostic tests would you recommend?”

Expected response: Consider ovarian cancer, benign ovarian cysts, and gastrointestinal issues. Recommended tests include serum CA-125, transvaginal ultrasound, and abdominal/pelvic CT scan.

##### 3.5.5.2 Multidisciplinary insights

Radiologist: Discusses ultrasound findings and malignant features such as solid components.

Pathologist: Explains the relevance of CA-125 levels and diagnostic protocols.

Oncologist: Provides insights on treatment strategies, discussing the roles of surgery and chemotherapy.

##### 3.5.5.3 Treatment plan development and ethical considerations

Question: “Based on the diagnostic findings, what would be your treatment approach?”

Expected response: Discuss neoadjuvant chemotherapy, exploratory laparotomy, and cytoreductive surgery options.

Ethical discussion: Students discuss how to communicate risks and benefits to the patient, considering informed consent and patient autonomy.

##### 3.5.5.4 Reflection on multidisciplinary collaboration

Question: “How did each discipline contribute to the overall management plan, and what value does this collaborative approach add?”

Expected discussion: Emphasizes the role of teamwork in accurate diagnosis, comprehensive care, and holistic treatment planning.

## 4 Discussion

The results of this study indicate that the MDT combined with CBL teaching model presents clear advantages over traditional teaching methods in gynecological oncology training. The new teaching model promotes a competency-based, student-centered approach, enhancing student engagement and learning outcomes. Interns in the research group rated the new model more favorably than the traditional approach, and their performance in terms of knowledge acquisition, clinical reasoning, teamwork, and communication skills showed statistically significant improvement compared to the control group.

This teaching method not only ignores the individual students’ independent learning potential and learning enthusiasm, but also limits students’ innovative thinking and critical thinking ability, affecting the quality of clinical skills training, and thus affecting the overall quality and effect of clinical teaching. Gynecological oncology is a highly professional branch of medicine. On the one hand, its practicality requires medical students to not only master solid theoretical basic knowledge but also have the practical ability to flexibly apply this knowledge in clinical practice (27). On the other hand, the reason why this subject is difficult to master is largely because it is closely related to the patient’s sensitive privacy information, which affects students’ interest and enthusiasm in this subject and makes learning more difficult. Therefore, it is particularly urgent to change the traditional teaching model and adopt more humane, diversified, and interactive educational methods.

As early as 1997, the University of Texas MD Anderson Cancer Center in the United States pioneered the MDT approach in the field of tumor treatment. Medical education scholars later applied this method to clinical teaching, involving joint teaching with instructors from multiple related disciplines (28). CBL is a teaching method that uses real clinical scenarios to prepare students for clinical practice (29). Unlike traditional textbook-style lectures, CBL encourages students to engage in peer learning and apply new knowledge to real clinical problems under the guidance of mentors (17, 30).

Compared to traditional teaching methods, CBL demonstrates unique advantages. Rather than merely imparting knowledge, it emphasizes driving the learning process through practical and meaningful results. This teaching model focuses on cultivating students’ rigorous logical reasoning abilities, enabling them to think independently and solve complex problems. CBL promotes a structured and critical approach to solving clinical problems (31). Trainees explore clinically relevant topics with clear goals under open-ended questions, discussing medical history, physical examination findings, and laboratory test results. Through the discussion of clinical cases related to the topics taught, students use higher-order cognition to assess their understanding of concepts, which encourages active learning and enhances critical thinking skills (32, 33).

The design of this study was to apply a teaching model combining multidisciplinary diagnosis and CBL teaching to the teaching of gynecological oncology interns in our hospital, to comprehensively assess the value of the clinical application of this model in the training of gynecological oncology interns. The results indicated that the overall evaluation of the teaching effect under the new model by teaching instructors was superior to that of the traditional teaching model. Interns in the research group also rated the new model more favorably than the traditional teaching approach. The research group outperformed the control group in terms of knowledge acquisition ability, learning initiative, learning interest, clinical integration, clinical analysis ability, clinical diagnosis and treatment thinking, teamwork ability, literature retrieval and reading ability, doctor-patient communication ability, and clinical language expression ability. The differences were statistically significant. The final examination scores, based on three dimensions—basic theoretical knowledge, clinical skills, and case analysis—were higher in the research group than in the control group, with significant differences observed. This indicates that the new model significantly stimulated the students’ potential for independent learning, consistent with findings from foreign literature reviews (5, 6, 15, 34, 35).

The new model overcomes the limitations of traditional teaching methods in undergraduate medical education by enhancing dynamic interaction between teachers and students. This dynamic learning process increases students’ interest and motivation, leading to greater concentration during lessons and maximizing learning potential. Students are encouraged to work in groups to deeply explore targeted cases designed by their instructors. They combine what they have learned and, under the guidance of multidisciplinary instructors, view clinical cases from multiple perspectives, analyze and deduce, and ultimately solve problems (24, 36). This approach not only improves their ability to analyze and solve problems and think comprehensively but also promotes mutual learning and collaboration among them. Such a communication platform allows students to share their ideas and experiences, and teachers to better understand each student’s characteristics and needs, providing personalized guidance and support, thereby enhancing student-teacher communication (37, 38).

Although planning MDT combined with CBL may require considerable time, especially in preparing clinical cases and coordinating with other instructors, teachers provided positive feedback in their self-assessment reports. The multidisciplinary lead teachers expressed hope that by introducing more practical cases and discussions, students would better understand how to balance accessing medical information with protecting patient privacy, thus increasing their interest and motivation in the subject. Through interdisciplinary team collaboration and guidance, we break down disciplinary barriers, promote the exchange and sharing of knowledge, and provide students with broader perspectives and deeper learning experiences. This improves the comprehensive diagnosis and treatment capabilities of interns, better preparing them for real-world medical and clinical challenges.

In summary, the MDT combined with the CBL teaching model, based on real clinical cases in gynecological oncology teaching, presented significantly better teaching effects than traditional methods. This aligns with the expectations of both teachers and students and serves as a major guarantee for the promotion of the new model.

## Data Availability

The original contributions presented in the study are included in the article/supplementary material, further inquiries can be directed to the corresponding author.
