# Supplementary material for: Application value of multi-disciplinary collaborative diagnosis and treatment combined with CBL teaching model in gynecological oncology practice teaching
Source: Front Med (Lausanne). 2025 Jan 8;11:1468256. doi: 10.3389/fmed.2024.1468256 (PMC11750869; doi:10.3389/fmed.2024.1468256)
Supplement: Supplementary file 1 [file Data_Sheet_1.docx]

**Questionnaire**

**Teacher Self-Assessment Questionnaire**

1. Does this pedagogical approach facilitate students' construction of theoretical frameworks and retention of key knowledge points?
2. Are students able to adequately complete pre-class preparation as assigned?
3. Are you, as the instructor, able to fully engage in classroom discussions?
4. Does the lesson planning process significantly increase the teacher's workload?
5. Does this pedagogical approach contribute to the enhancement of the teacher's own knowledge and skills?
6. Has there been a noticeable improvement in students' practical competencies?

**Student Survey of Pedagogical Model Acceptance**

1. Ability to acquire knowledge
2. Enhancement of learning initiative
3. Stimulation of interest in the subject matter
4. Increased integration of clinical knowledge
5. Development of clinical analysis skills
6. Enhancement of clinical diagnostic reasoning
7. Improvement in teamwork and collaboration skills
8. Enhancement of literature search and critical appraisal skills
9. Improvement in doctor-patient communication skills
10. Refinement of clinical language and expression skills
